# Verbascoside Inhibits/Repairs the Damage of LPS-Induced Inflammation by Regulating Apoptosis, Oxidative Stress, and Bone Remodeling

**DOI:** 10.3390/cimb45110550

**Published:** 2023-10-31

**Authors:** Sahika Pinar Akyer, Ege Rıza Karagur, Melek Tunc Ata, Emine Kilic Toprak, Aysegul Cort Donmez, Baris Ozgur Donmez

**Affiliations:** 1 Department of Anatomy, School of Medicine, Pamukkale University, Kinikli, Str. No. 11, 20160 Denizli, Turkey; spakyer@pau.edu.tr; 2 Department of Medical Genetics, School of Medicine, Pamukkale University, Kinikli, Str. No. 11, 20160 Denizli, Turkey; ekaragur@pau.edu.tr; 3 Department of Physiology, School of Medicine, Pamukkale University, Kinikli, Str. No. 11, 20160 Denizli, Turkey; melekt@pau.edu.tr (M.T.A.); ektoprak@pau.edu.tr (E.K.T.); 4 Department of Medical Biochemistry, School of Medicine, Pamukkale University, Kinikli, Str. No. 11, 20160 Denizli, Turkey; aysegulcort@pau.edu.tr

**Keywords:** MLO-Y4 cell, bone metabolism, verbascoside, LPS, bone proteins

## Abstract

Osteocytes play an important role as regulators of both osteoclasts and osteoblasts, and some proteins that are secreted from them play a role in bone remodeling and modeling. LPS affects bone structure because it is an inflammatory factor, despite verbascoside’s potential for bone preservation and healing. Osteocytes may also be involved in the control of the bone’s response to immunological changes in inflammatory situations. MLO-Y4 cells were cultured in either supplemented -MEM alone with a low serum to inhibit cell growth or media with LPS (10 ng/mL) and/or verbascoside (50 g/mL) to show the LPS effect. In our research, LPS treatment increased RANKL levels while decreasing OPG and RUNX2 expression. Treatment with verbascoside reduced RANKL expression. In our work, verbascoside increased the expression of OPG and RUNX2. In MLO-Y4 cells exposed to verbascoside, SOD, CAT, and GSH activities as well as the expression levels of bone mineralization proteins like PHEX, RUNX2, and OPG were all elevated.

## 1. Introduction

The main structural element of the skeletal system that allows for movement, muscle attachment, organ protection, and calcium balance is bone. Additionally, bone creates a special milieu for osteoblasts, osteoclasts, and osteocytes [1]. The primary bone cells involved in bone formation and bone resorption are osteoblasts and osteoclasts. The other cell is osteocytes, which are embedded in the mineralized bone matrix [2]. The majority of bone cells (90–95%) are osteocytes [3], and osteocytes continue to increase with age and bone size [4]. A number of disorders can cause an imbalance between bone growth and resorption, which results in bone loss, such as aging and some inflammatory conditions [5], rheumatoid arthritis [6], periodontitis [7], obesity [8], and diabetes [9]. On the other hand, increased fracture incidence is also linked to inflammatory diseases [10,11]. Osteocytes are now recognized as important regulators of both osteoclasts and osteoblasts, directing changes in bone turnover, because of their responsiveness to inflammatory stimuli [5,12]. Pro-inflammatory cytokines are released by osteocytes in response to inflammation, and these cytokines both autocrinely and paracrinely modify the function of bone cells. The osteocyte can exacerbate inflammation in addition to acting as a bone cell or an endocrine cell. As a result, treating inflammation-related bone loss and other clinical consequences may involve targeting the osteocyte as a potential therapeutic target [13]. Another significant link between postmenopausal osteoporosis and oxidative stress is inflammation. The body experiences chronic inflammation as a result of oxidative stress, which damages biomolecules and produces chemokines and cytokines that attract and activate inflammatory cells [14]. Because osteocytes also express some important proteins such as dentin matrix protein 1 (DMP1), phosphate-regulating gene with homologies to endopeptidases on the X chromosome (PHEX), matrix extracellular phosphoglycoprotein (MEPE), Runt-related transcription factor-2 (RUNX2), Osteoprotegerin (OPG), receptor activator of nuclear factor kappa-Β (RANK) and its ligand (RANKL) play a role in mineral homeostasis [15,16]. Phosphate-regulating neutral endopeptidase on chromosome X (PHEX) and dentin matrix protein 1 (DMP1) are the most important markers for osteocytes. When it comes to mature osteocytes, some of these markers include matrix extracellular phosphoglycoprotein (MEPE), SOST/sclerostin, and receptor activator of nuclear factor-κB ligand (RANKL). PHEX, DMP1, MEPE, and FGF23 are a few of the markers that are involved in mineralization and phosphate homeostasis. Osteocytes regulate the remodeling of the skeleton by secreting RANKL, an activator of osteoclast function, and sclerostin, a negative regulator of osteoblastic bone production [17]. Osteocytes have a key role in the balance of osteoblast and osteoclast regulation, which contains multiple proteins. Some of these proteins are secreted by osteocytes and one of their main roles is to provide bone modeling and remodeling. Sclerostin, which is expressed and secreted in osteocytes and other terminally differentiated cell types embedded inside mineralized matrices, suppresses osteoblastic bone production, and PHEX, another osteocyte-specific protein, is linked to biomineralization and phosphate homeostasis [18,19,20]. MEPE has an important role in bone mineralization and maintains the balance of mineralization in the osteocyte environment, especially against mechanical loading [21]. When osteoblasts and osteocytes adhere to RANK on osteoclast progenitor cells, which eventually give rise to osteoclasts, they create RANKL and OPG. Additionally, cells in the osteoblast lineage produce osteoprotegerin (OPG), which inhibits RANKL from binding to RANK.

Additionally, these osteocyte-secreted proteins play a crucial part in bone modeling and remodeling [22]. Verbascoside is also called acteoside, which is one of the phenylethanoid glycoside components in some medicinal plants such as Verbascum and Cistanche [23]. In recent years, there are some studies showing the mechanism of action of acteoside as antioxidant [24], anti-inflammatory [25], and anti-osteoporotic [26,27,28]. Li et al. showed that *C. deserticola* extract induces osteoblast differentiation and maturation but not proliferation or migration and its increased bone mineralization [27]. In another study showing the relationship between asteoside and osteocyte, Lee et al. showed that acteoside inhibits RANKL-induced osteoclast differentiation and suppresses bone resorption by mature osteoclasts [29]. Xu et al. showed that cistanoside A, which is an active phenylethanoid glycoside isolated from *C. deserticola*, had significant antiosteoporotic activity on ovariectomized (OVX) mice, and they indicated that there was increased bone strength in OVX mice treated with different doses of cistano-side A and improvement in bone quality [28]. An important reaction to injury or infection is inflammation, which alerts the immune system to repair damaged tissues or defend the body from invading pathogens, although low levels of circulating pro-inflammatory cytokines are frequently indicative of chronic low-grade inflammation [30,31]. Gram-negative bacteria have a substance which is called lipopolysaccharide (LPS) on their outer membranes. These bacteria have the ability to release LPS, with small amounts entering the bloodstream normally through the gut epithelium [30]. Some of the previous studies showed that trabecular bone structures, bone mineral density, and bone strength were adversely affected by LPS treatment [32,33,34,35,36,37]. Therefore, inflammatory effects and genotoxic stress may occur in osteocyte cells exposed to LPS for a long time.

All of the above evidence suggests that verbascoside may have bone-preserving and bone-repairing properties, but LPS damages bone structure as an inflammatory factor. However, there are no studies in the literature describing the relationship between verbascoside and MLO-Y4 cells under the inflammation conditions. The regulation of the bone’s reaction to immunological alterations in inflammatory conditions may also be carried out by osteocytes. It is clear that osteocytes react to inflammatory signals in many ways, such as by balancing their expression of PHEX, MEPE, RANKL, RUNX2, and OPG. In the present study, it was aimed to explain the mechanism of verbascoside, which has a positive effect on bone tissue, on MLO-Y4 cells. Our hypothesis is that LPS may change the expression amount of bone mineralization proteins in MLO-Y4 cells, but verbascoside may have a regulatory effect on the amount of proteins released from osteocytes like PHEX, MEPE, RANKL, RUNX2, and OPG.

## 2. Material and Methods

### 2.1. Cell Culture and Viability

MLO-Y4 cells were obtained from the Kerafast company. MLO-Y4 cells were cultured in type I rat tail collagen-coated cell culture dishes at 37 °C in a humidified atmosphere containing 5% CO_2_ air in a completed medium (%89 alpha-minimum essential medium (α-MEM), 5% fetal bovine serum; 5% calf serum and 1% penicillin-streptomycin). To evaluate the LPS effect, cells were cultured in supplemented α-MEM alone containing low serum (2% FBS) to reduce cell proliferation or media containing LPS (10 ng/mL) and/or verbascoside (50 μg/mL). Cell viability was determined using water-soluble tetrazolium salt (WST)-8 reagent. WST-8 reagent was added into the cultures after 48 h of incubation. After incubating for an additional 4 h, the absorbance was measured at 450 nm using a microplate reader (ThermoFisher, Vantaa, Finland).

### 2.2. CAT, SOD, GSH, NOX and Caspase-3 Measurements

MLO-Y4 cells (1 × 10^6^ cells) were seeded in 6-well plates and maintained for 3 days for the proper attachment, and incubated with verbascoside during 24 h. At the end of the time point, cells were washed with PBS and collected and lysed with ultrasonic wave treatment. After centrifugation at 2000× *g* for 5 min, the supernatant fractions were collected and measurements were conducted. Superoxide dismutase (SOD), catalase (CAT), glutathione (GSH), nitric oxide (NOX), and caspase-3 content were determined using corresponding colorimetric diagnostic kits (Shanghai YL Biotech, Shanghai, China). Cell lysates and HRP-conjugate reagent were added to the ELISA plate and incubated at 37 °C for 60 min. The liquid was discarded and the ELISA plate was washed five times with the wash buffer. Next, chromogen solution was added to each well and avoided the light for 15 min at 37 °C. Then, stop solution was added to each well, indicated by the blue color changing to yellow. Finally, the OD value of each well was measured at 450 nm and converted to a standard curve. Protein amounts of the samples were measured according to the Bradford Method.

### 2.3. Determination of the Total Antioxidant Status, Total Oxidant Status, and Oxidative Stress Index

Total antioxidant status (TAS) and total oxidant status (TOS) were measured in MLO-Y4 cells using the test kit from Rel Assay Diagnostics according to the manufacturer’s instructions. Oxidative Stress Index (OSI) was measured as: OSI = [(TOS, μmol H_2_O_2_ equivalent/L)/(TAS, mmol Trolox equivalent/L) × 10]. Total antioxidant capacity (TAC) and total oxidant status (TOS) rapid and reliable automated colorimetric assays are frequently used to determine the oxidative alterations. The results were considered as mM Trolox equivalent per liter. The oxidative stress index (OSI) was found by dividing the TOS level by the TAC level [38].

### 2.4. RNA Isolation, cDNA Synthesis, and Quantitative Real Time Polymerase Chain Reaction (qRT-PCR)

The mRNA expression of bone remodeling markers, such as MEPE, PHEX, RANKL, RUNX2, and OPG was determined by qRT-PCR (Table 1). In brief, total RNA was extracted from cells with Geneall kit (GeneAll, Seoul, Republic of Korea) according to the manufacturer’s instructions. OneScript Plus cDNA Synthesis Kit was utilized for the synthesis of cDNA (abm, Richmond, BC, Canada). Also, mRNA expression levels were determined by using SybrGreen mRNA qPCR MasterMix. Bio-Rad CFX96 was used for all qRT-PCR reactions. All PCR reactions were performed at least in triplicate, and the expression levels were normalized to the endogenous control; GAPDH was used for mRNA quantification. According to the manufacturer’s protocol, each technique was carried out. Data were analyzed using the 2^−ΔΔCt^ method.

### 2.5. Statistical Analysis

All the data were given as the average ± standard error (mean ± SE). Statistical analysis was performed using SPSS packed program for Windows version 10.0 (SPSS Inc., Chicago, IL, USA). *p* < 0.05 was considered as a significant difference. Comparison of the non-parametric data among the groups was performed using the Mann–Whitney *U* test.

## 3. Results

### 3.1. The Effects of Verbascoside Cell Viability in MLO-Y4 Cells

**Figure 1:**
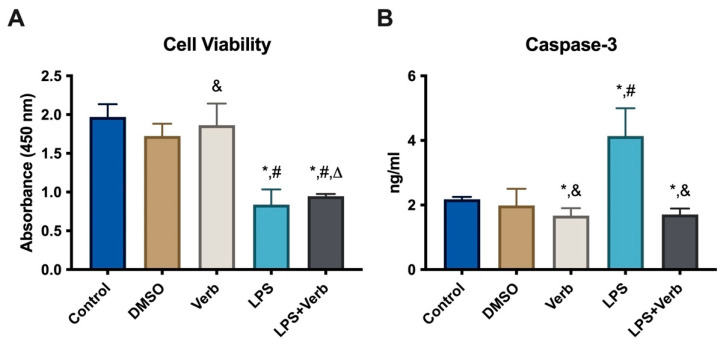
Verbascoside increases cell viability by suppressing caspase-3 in MLO-Y4 cells. (**A**) Cell viability of MLO-Y4 cells incubated with DMSO (used as vehicle), 50 μg/mL Verb, 100 ng/mL LPS, LPS + Verb for 48 h. Cell viability was measured using WST-8 regent (*n* = 5 per each experiment). * *p* < 0.001 vs. control. # *p* < 0.001 vs. DMSO. & *p* < 0.001 vs. LPS. Δ *p* < 0.001 vs. Verb. (**B**) Caspase-3 levels in MLO-Y4 cells incubated with DMSO (used as vehicle), 50 μg/mL Verb, 100 ng/mL LPS, LPS + Verb for 48 h. * *p* = 0.002 cells treated with LPS, Verb, LPS + Verb vs. untreated control cells, # *p* < 0.001 LPS vs. DMSO, & *p* < 0.001 Verb and LPS + Verb vs. LPS (*n* = 5). LPS, lipopolysaccharide; Verb, verbascoside.

To verify the effect of verbascoside on MLO-Y4 cell viability in inflammatory conditions, the cells were cultured with 50 μg/mL concentration of verbascoside and/or 100 ng/mL LPS for 48 h. Verbascoside did not change the number of osteocytes alone. LPS treatment led to 57.5% reduction in the number of viable cells. When the cells were treated concomitantly with verbascoside and LPS, the osteocyte number increased by 6% compared to cells treated with LPS alone (Figure 1A). Caspase-3 levels were evaluated in MLO-Y4 cells grown in media supplemented with 10 ng/mL LPS and/or 50 μg/mL verbascoside for 48 h (Figure 1B). Consistent with this result, verbascoside treatment decreased the cell LPS depending on the cell death of osteocytes. Caspase-3 level was significantly elevated by the LPS incubation compared to untreated control cells and the dimethyl sulfoxide (DMSO)-treated cell group. Treatment with verbascoside alone led to a significant decrease in caspase-3 levels compared to untreated control cells. The concomitant incubation of verbascoside and LPS significantly decreased the caspase-3 levels compared to single LPS treatment. The data obtained from our results show that LPS significantly activates the apoptosis in MLO-Y4 cells. This activation is suppressed by the verbascoside treatment. 

### 3.2. Verbascoside Regulated the Activity of Oxidant and Antioxidant

**Figure 2:**
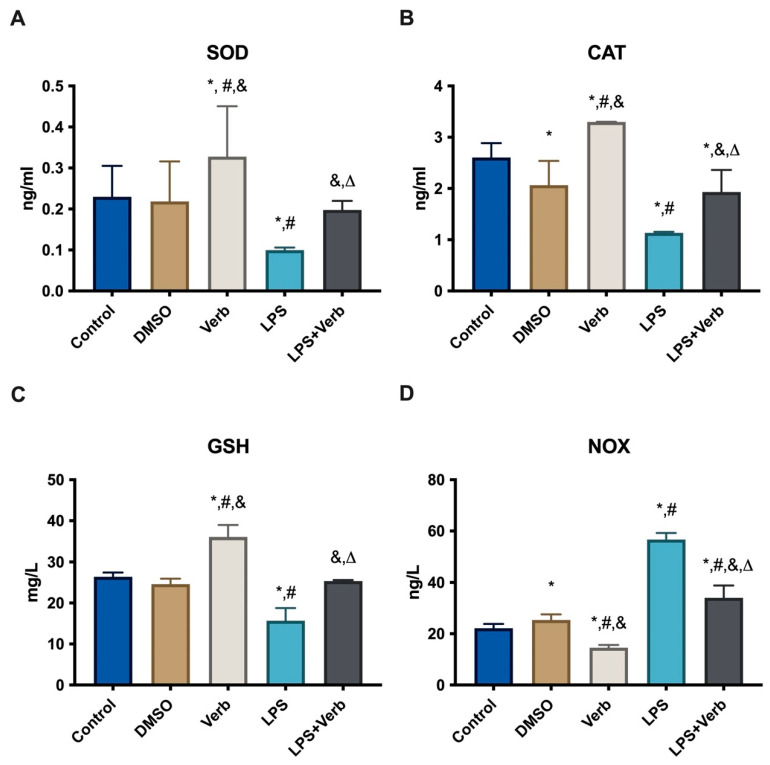
Effects of verbascoside on SOD, CAT, GSH and NOX in MLO-Y4 cells. (**A**). SOD levels in MLO-Y4 cells incubated with DMSO (used as vehicle), 50 μg/mL Verb, 100 ng/mL LPS, LPS + Verb for 48 h. * *p* = 0.001 cells treated with LPS and Verb vs. untreated control cells, # *p* = 0.0015 LPS and Verb vs. DMSO, & *p* = 0.0148 Verb and LPS + Verb vs. LPS, Δ *p* < 0.001 LPS + Verb vs. Verb (*n* = 5). (**B**). CAT levels in MLO-Y4 cells incubated with DMSO (used as vehicle), 50 μg/mL Verb, 100 ng/mL LPS, LPS + Verb for 48 h. * *p* < 0.0001 cells treated with DMSO, LPS, Verb, LPS + Verb vs. untreated control cells, # *p* < 0.001 LPS and Verb vs. DMSO, & *p* < 0.001 Verb and LPS + Verb vs. LPS, Δ *p* < 0.001 LPS + Verb vs. Verb (*n* = 5). (**C**). GSH levels in MLO-Y4 cells incubated with DMSO (used as vehicle), 50 μg/mL Verb, 100 ng/mL LPS, LPS + Verb for 48 h. * *p* < 0.0001 cells treated with LPS, Verb vs. untreated control cells, # *p* < 0.001 LPS and Verb vs. DMSO, & *p* < 0.001 Verb and LPS + Verb vs. LPS, Δ *p* < 0.001 LPS + Verb vs. Verb (*n* = 5). (**D**). NOX levels in MLO-Y4 cells incubated with DMSO (used as vehicle), 50 μg/mL Verb, 100 ng/mL LPS, LPS + Verb for 48 h. * *p* < 0.0001 cells treated with DMSO, LPS, Verb, LPS + Verb vs. untreated control cells, # *p* < 0.001 LPS, Verb, LPS + Verb vs. DMSO, & *p* < 0.001 Verb and LPS + Verb vs. LPS, Δ *p* < 0.001 LPS + Verb vs. Verb (*n* = 5). LPS, lipopolysaccharide; Verb, verbascoside.

Age-related diseases like osteoporosis can be impacted by oxidative stress, which also has a major impact on osteocytes. Therefore, oxidative parameters such as SOD, CAT, GSH, and NOX are very important to demonstrate this relationship. SOD levels were evaluated in MLO-Y4 cells grown in media supplemented with 10 ng/mL LPS and/or 50 μg/mL Verb for 48 h (Figure 2A). SOD level was elevated by the Verb incubation compared to untreated control cells and the DMSO-treated cell group. Treatment with LPS alone led to a significant decrease in SOD levels compared to untreated control cells. The concomitant incubation of Verb and LPS significantly elevated the SOD levels compared to single LPS treatment. The data obtained from our results show that Verb significantly inhibited the effects of LPS on SOD activity in MLO-Y4 cells. CAT levels were evaluated in MLO-Y4 cells grown in media supplemented with 10 ng/mL LPS and/or 50 μg/mL verbascoside for 48 h (Figure 2B). The CAT level was significantly elevated by the verbascoside incubation compared to the untreated control cells and DMSO-treated cell group. Treatment with LPS led to a significant decrease in CAT levels compared to untreated control cells. The concomitant incubation of verbascoside and LPS significantly enhanced the CAT levels compared to single LPS treatment. GSH levels were evaluated in MLO-Y4 cells grown in media supplemented with 10 ng/mL LPS and/or 50 μg/mL verbascoside for 48 h (Figure 2C). GSH level was significantly elevated by the verbascoside incubation compared to the untreated control cells and DMSO-treated cell group. Treatment with LPS led to a significant decrease in GSH levels compared to untreated control cells. The concomitant incubation of verbascoside and LPS significantly enhanced the CAT levels compared to single LPS treatment. NOX levels were evaluated in MLO-Y4 cells grown in media supplemented with 10 ng/mL LPS and/or 50 μg/mL verbascoside for 48 h (Figure 2D). NOX level was significantly elevated by LPS incubation compared to untreated control cells and the DMSO-treated cell group. Treatment with verbascoside alone led to a significant decrease in NOX levels compared to untreated control cells. The concomitant incubation of verbascoside and LPS significantly decreased the NOX levels compared to single LPS treatment. The data obtained from our results show that LPS significantly activates the NOX in MLO-Y4 cells. This activation is suppressed by the verbascoside treatment. 

**Figure 3:**
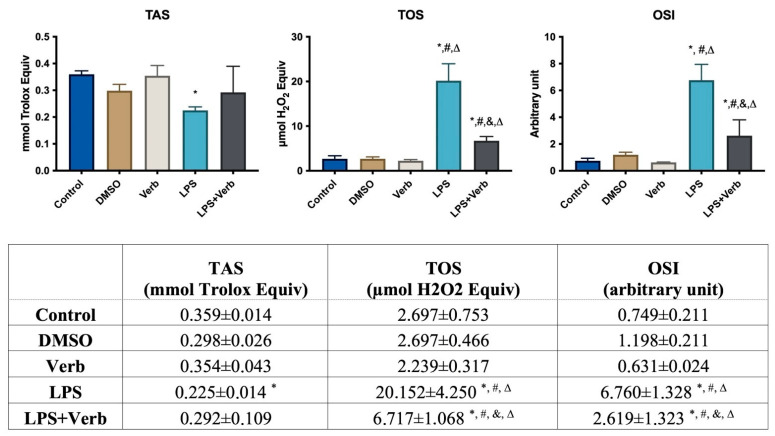
Oxidative stress parameters of MLO-Y4 cells incubated with verbascoside. 50 μg/mL Verb, 100 ng/mL LPS, LPS + Verb for 48 h. * *p* < 0.05 vs. control, # *p* < 0.05 vs DMSO, & *p* < 0.05. vs. LPS, Δ *p* < 0.05 vs Verb. TOS, total oxidant status; TAS, total antioxidant status; OSI, oxidative stress index.

The effects of verbascoside and/or LPS for 48 h on oxidative stress were determined in MLO-Y4 cells by using the TAS-TOS assay. TAS levels significantly decreased in MLO-Y4 cells treated with LPS compared to untreated control cells. The TOS levels significantly increased in MLO-Y4 cells treated with LPS and LPS + Verb groups. OSI levels were elevated by LPS and its verbascoside combination (Figure 3).

### 3.3. Verbascoside Regulates the Balance of Mineralization and Demineralization via PHEX, RUNX2, OPG, RANKL, MEPE Genes

**Figure 4:**
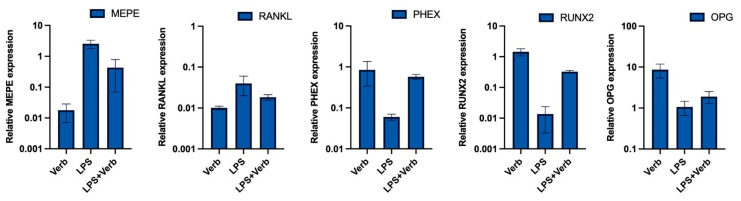
The verbascoside regulates bone formation by balancing osteogenic markers. The graphics show mRNA expression alteration of MEPE, RANKL, PHEX, RUNX2, OPG after verbascoside and/or LPS treatment. The results were presented as fold changes according to the 2^−ΔΔCt^ method. DMP1: dentin matrix protein 1, PHEX: phosphate-regulating gene with homologies to endopeptidases on the X chromosome, MEPE: matrix extracellular phosphoglycoprotein, RUNX2: Runt-related transcription factor-2, OPG: Osteoprotegerin, RANKL: Receptor activator of nuclear factor kappa-Β ligand.

qRT-PCR analyses showed that PHEX, RUNX2, and OPG gene expression in MLO-Y4 cell cultures was affected by the presence or not of LPS and verbascoside. Verbascoside dramatically increased the expression levels of PHEX, RUNX2, and OPG. The levels of expression of PHEX and RUNX2 were upregulated in cultures exposed to LPS + verbascoside and verbascoside in comparison to the cell group treated with LPS alone (Figure 4). The peak levels of PHEX, RUNX2, and OPG expression were determined in single verbascoside treatment. Concomitant verbascoside treatment with LPS led to a significant decrease in MEPE and RANKL expression levels compared to LPS treatment alone. The deepest levels of MEPE and RANKL expression were observed in the single treatment of verbascoside. 

## 4. Discussion

Osteocytes are an important cell type that is abundant in bone tissue and manages the balance between osteoblasts and osteoclasts. Basically, mechanisms targeting osteocytes often disrupt this balance, resulting in osteoporosis, which disrupts bone microarchitecture and damages bone. The MLO-Y4 cell line, which is a cell line with an osteocyte-like phenotype, represents a suitable model for studying the biological functions and properties of the osteocytes. The excess inflammatory response, oxidative stress, and apoptotic cell death were considered as the main culprits responsible for inflammation and Reactive Oxygen Species (ROS)-induced bone loss. It has been reported that some natural products provide healing in bone tissue by reducing oxidative stress in diseases that damage bone tissue, especially diseases such as osteoporosis and osteoarthritis. Inflammation, which is one of the most important responses to damage in tissue, this process continues with the expression of mediators including cytokines, chemokines, and ROS [29]. Verbascoside has some important antioxidant effects that occur in various cell systems, and different mechanisms of action have been identified for these. The first is short-term ROS scavenging by interfering with or preventing the initial ROS-producing reactions that indicate ROS-related damage. Another mechanism is to scavenge free oxygen molecules required to initiate ROS production and create long-term genomic effects for the down-regulation of genes encoding pro-oxidant enzymes [39,40]. Some of the in-vivo studies have shown relationships between LPS and bone structures; deterioration of bone structure has been revealed with LPS exposure [19,32,34,35,36]. Droke et al. showed that negative effects of LPS induced alterations in bone structure, and these effects upregulated the TNF-α expression in the tibia metaphyseal region [32]. In a different study, male mice exposed to LPS for four weeks had decreased bone mineral density of the vertebral body and bone volume/total volume ratio, which affected bone strength [19]. In a similar study, Cao et al. showed that female mice exposed to LPS for 13 weeks developed damaged bone structure in the distal femur and second lumbar vertebra, and it was caused by increased osteoclastogenesis and decreased bone formation [34]. Smith et al. revealed that reduced bone volume total volume ratio, decreased trabecular number, and increased trabecular separation as a result of the overexpression of cytokines like IL-1 and TNF- by LPS led to impaired bone strength [35]. Similarly, Shen et al. showed a decrease in femur bone mineral density with twelve weeks of LPS exposure in old female rats [36]. The goal of this work was to evaluate the orchestrator effects of verbascoside on LPS-induced inflammation as a bone resorption model in osteocyte cells.

**Figure 5:**
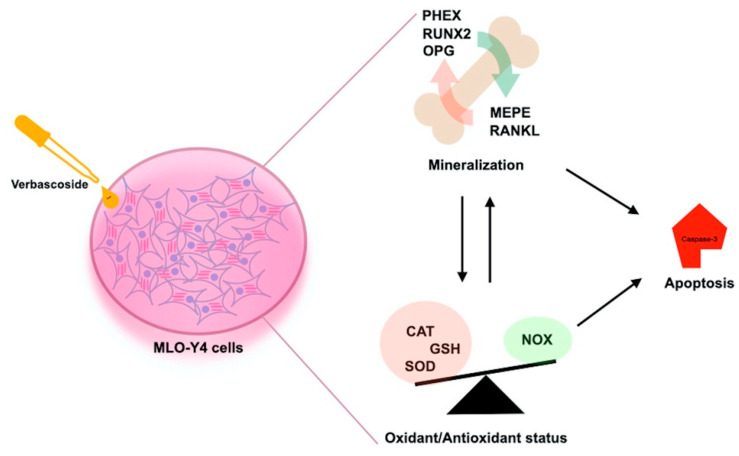
Hypothesized schematic model explaining molecular mechanisms how verbascoside regulate bone remodeling.

In inflammation, the levels of pro-inflammatory cytokines increase, which leads to apoptosis of osteocyte cells, which causes the release of many inflammatory cytokines and signaling molecules that disrupt distant organ function. As a result of this imbalance caused by apoptosis, bone-related diseases such as osteonecrosis, osteoporosis, and osteoarthritis may occur [5]. Our results indicated that the caspase 3 level as an apoptosis marker was significantly increased by LPS. Anti-apoptotic strategies include inhibition of protease enzymes like caspase-3. Our results indicated that verbascoside (50 μg/mL) has a suppressor effect on apoptosis by inhibiting the caspase-3 without affecting cell viability (Figure 1 and Figure 3). Oxidative stress and inflammation in postmenopausal osteoporosis has been related to the activation of NADPH oxidase and/or decreased synthesis of SOD, CAT, and GSH levels [41]. Superoxide oxidoreductase (SOD) is the first defense mechanism of antioxidant enzymes, and catalyzes the dismutation reaction to convert superperoxide radical anion (^•^O^−2^) into hydrogen peroxide (H_2_O_2_) [39]. On the other hand, it has also been shown that increasing mitochondrial SOD activity prevents ROS-induced osteoblast apoptosis. [40]. The antioxidant mechanism of CAT, which is the second defense system of antioxidant enzymes, is mainly to affect the dismutation reaction on (H_2_O_2_) produced in SOD-mediated processes [42]. CAT can have a positive effect on bone mass by inhibiting H_2_O_2_-induced osteoclastic resorption [43]. According to our results, verbascoside treatment resulted in increased activity of SOD and CAT, which are inhibited by LPS. Elevated activity of SOD and CAT by the force of verbascoside prevents the osteocytes from the deteriorative effects of inflammation. Additionally, verbascoside exerts its anti-apoptotic effects by decreasing caspase-3 activity in MLO-Y4 cells. Our results prove the supportive role of verbascoside on bone mineralization through regulating the balance between SOD, CAT, and caspase-3 (Figure 2, Figure 3 and Figure 4). Free radicals lose their electronegativity by taking a pair of electrons from the sulfhydryl group (-SH) in GSH, thus losing their strong oxidizing and aggressive properties [44]. Verbascoside treatment supported the antioxidant activity by increasing GSH levels in osteocytes (Figure 5). OSI was utilized as an indicator of an antioxidant/oxidant system in our study. While LPS increased the OSI levels significantly, verbascoside decreased the OSI levels alone or in combination with LPS (Table 1). NADPH oxidases, as one of the most important sources of ROS, have been studied quite deeply for their role in osteocyte remodeling [45]. LPS stimulation resulted in activated NOX levels. Verbascoside not only has a preventive effect, but also has a supportive effect on bone structure (Figure 4). Inflammatory signals also influence osteocyte proteins controlling bone formation. In our study we have investigated the expression levels of bone remodeling markers such as PHEX, MEPE, RUNX2, RANKL, and OPG. The osteocyte microenvironment is modulated by mineralization via one of the most important bone mineralization regulators PHEX and MEPE. It was shown that osteoblast number and activity were significantly enhanced, leading to a rise in bone mass in MEPE knockout mice [46]. Additionally, increased MEPE protein production in the bone causes a deficit in mineralization in a murine mouse model [47]. PHEX is produced and released in osteocytes and other terminally differentiated cell types embedded inside mineralized matrices, and it is exclusive to osteoclasts [18]. Understanding the metabolism of these proteins is crucial because PHEX and MEPE depletion or inactivation can lead to some serious clinical issues. Donmez BO et al. suggest that different calcium concentrations can trigger bone mineralization proteins such as PHEX, MEPE, and DMP1 in osteocytes [48]. Our results showed that there is an antagonistic behavioral modulation between the MEPE and PHEX correlated with previous studies. Verbascoside treatment inhibited the MEPE expression and induced the PHEX expression in MLO-Y4 cells. LPS-induced bone resorption could be prevented by verbascoside via the PHEX and MEPE crosstalk. Verbascoside exerts its multiple biological functions by controlling the expression of different molecules as well as by modulating different signaling pathways. The osteoblasts release OPG, which binds to RANKL to prevent the osteoclast precursor from differentiating into a mature osteoclast, thus inhibiting the formation of osteoclastic materials [49]. Levels of the OPG and RANKL are used as an indicator of osteoclastogenesis. In our experiments, LPS treatment elevated the level of RANKL and downregulated the expression of OPG and RUNX2. It was shown that pretreatment with antioxidants inhibits RANKL-induced activation of NF-kB, thereby suppressing osteoclastogenesis. The RANKL/OPG/RUNX2 signaling axis has an important role in the regulation of bone remodeling. Verbascoside treatment decreased the RANKL expression in MLO-Y4 cells. While OPG and RUNX2 expression were stimulated by verbascoside in our study, SOD, CAT, and GSH activities and the expression levels of bone mineralization proteins such as PHEX, RUNX2, and OPG were increased in MLO-Y4 cell cultures exposed to verbascoside. More studies are needed to elucidate the mechanisms of action of verbascoside and to demonstrate its effects on human osteocyte cells. Taken together, these results suggested that verbascoside can positively regulate osteogenesis in vitro and prevent bone resorption induced by inflammation (Figure 5).

## Figures and Tables

**Table 1:** Primer sequences of RUNX2, OPG, RANKL, PHEX, MEPE, and GAPDH.

Gene	Forward Primer	Reverse Primer
*RUNX2*	GGCGTCAAACAGCCTCTTCA	GCTCACGTCGCTCATCTTGC
*OPG*	CGAGTGATGAATGCGTGTACTG	CTTCGCACAGGGTGACATCTATT
*RANKL*	AGCGAAGACACAGAAGCACTAC	TTTATGGGAACCCGATGGGATG
*PHEX*	TACTGCCTGAAGCCAGAATG	CCACAGAAAGATTTACTTTGCTCA
*MEPE*	ACTGTTCCTCTTCAGTATGAC	TGATATTTCTGAGGAGGGTG
*GAPDH*	AGGTCGGTGTGAACGGATTTG	GGGGTCGTTGATGGCAACA

## Data Availability

All raw data was used and analysed in the present study are available from the corresponding author of reasonable request.

## References

[1] Compton J.T., Lee F.Y. (2014). A review of osteocyte function and the emerging importance of sclerostin. J. Bone Joint. Surg Am..

[2] Palumbo C., Ferretti M. (2021). The Osteocyte: From “Prisoner” to “Orchestrator”. J. Funct. Morphol. Kinesiol..

[3] Bonewald L.F. (2011). The amazing osteocyte. J. Bone Miner. Res..

[4] Florencio-Silva R., da Silva Sasso G.R., Sasso-Cerri E., Simões M.J., Cerri P.S. (2015). Biology of Bone Tissue: Structure, Function, and Factors That Influence Bone Cells. BioMed Res. Int..

[5] Metzger C.E., Narayanan S.A. (2019). The Role of Osteocytes in Inflammatory Bone Loss. Front. Endocrinol..

[6] Pathak J.L., Bakker A.D., Luyten F.P., Verschueren P., Lems W.F., Klein-Nulend J., Bravenboer N. (2016). Systemic Inflammation Affects Human Osteocyte-Specific Protein and Cytokine Expression. Calcif. Tissue Int..

[7] Badran Z., Struillou X., Verner C., Clee T., Rakic M., Martinez M.C., Soueidan A. (2015). Periodontitis as a risk factor for systemic disease: Are microparticles the missing link?. Med. Hypotheses.

[8] Denis G.V., Sebastiani P., Bertrand K.A., Strissel K.J., Tran A.H., Slama J., Medina N.D., Andrieu G., Palmer J.R. (2018). Inflammatory signatures distinguish metabolic health in African American women with obesity. PLoS ONE.

[9] Tanaka K.-I., Yamaguchi T., Kanazawa I., Sugimoto T. (2015). Effects of high glucose and advanced glycation end products on the expressions of sclerostin and RANKL as well as apoptosis in osteocyte-like MLO-Y4-A2 cells. Biochem. Biophys. Res. Commun..

[10] van Staa T.P., Geusens P., Bijlsma J.W.J., Leufkens H.G.M., Cooper C. (2006). Clinical assessment of the long-term risk of fracture in patients with rheumatoid arthritis. Arthritis Rheum..

[11] Ogdie A., Harter L., Shin D., Baker J., Takeshita J., Choi H.K., Love T.J., Gelfand J.M. (2017). The risk of fracture among patients with psoriatic arthritis and psoriasis: A population-based study. Ann. Rheum. Dis..

[12] Kato Y., Windle J.J., Koop B.A., Mundy G.R., Bonewald L.F. (1997). Establishment of an osteocyte-like cell line, MLO-Y4. J. Bone Miner. Res..

[13] Zhou M., Li S., Pathak J.L. (2019). Pro-inflammatory Cytokines and Osteocytes. Curr. Osteoporos. Rep..

[14] Sindhu S., Akhter N., Wilson A., Thomas R., Arefanian H., Al Madhoun A., Al-Mulla F., Ahmad R. (2020). MIP-1α Expression Induced by Co-Stimulation of Human Monocytic Cells with Palmitate and TNF-α Involves the TLR4-IRF3 Pathway and Is Amplified by Oxidative Stress. Cells.

[15] Kulkarni R.N., Bakker A.D., Everts V., Klein-Nulend J. (2010). Inhibition of osteoclastogenesis by mechanically loaded osteocytes: Involvement of MEPE. Calcif. Tissue Int..

[16] Zhang C., Bakker A.D., Klein-Nulend J., Bravenboer N. (2019). Studies on Osteocytes in Their 3D Native Matrix Versus 2D In Vitro Models. Curr. Osteoporos. Rep..

[17] Robling A.G., Bonewald L.F. (2020). The Osteocyte: New Insights. Annu. Rev. Physiol..

[18] Guo D., Keightley A., Guthrie J., Veno P.A., Harris S.E., Bonewald L.F. (2010). Identification of osteocyte-selective proteins. Proteomics.

[19] Patalong-Wójcik M., Golara A., Zając K., Sokołowska A., Kozłowski M., Tołoczko-Grabarek A., Krzyścin M., Brodowska A., Janiec A., Myszka A. (2023). Influence of Muscle Mass and Strength on Bone Mineralisation with Consideration of Sclerostin Concentration. Biomedicines.

[20] Chin K.-Y., Ng B.N., Rostam M.K.I., Fadzil N.F.D.M., Raman V., Yunus F.M., Mark-Lee W.F., Chong Y.Y., Qian J., Zhang Y. (2023). Effects of E’Jiao on Skeletal Mineralisation, Osteocyte and WNT Signalling Inhibitors in Ovariectomised Rats. Life.

[21] Fisher L.W., Fedarko N.S. (2003). Six genes expressed in bones and teeth encode the current members of the SIBLING family of proteins. Connect. Tissue Res..

[22] Kao R.S., Abbott M.J., Louie A., O’Carroll D., Lu W., Nissenson R. (2013). Constitutive protein kinase A activity in osteocytes and late osteoblasts produces an anabolic effect on bone. Bone.

[23] Yang L., Zhang B., Liu J., Dong Y., Li Y., Li N., Zhao X., Snooks H., Hu C., Ma X. (2019). Protective Effect of Acteoside on Ovariectomy-Induced Bone Loss in Mice. Int. J. Mol. Sci..

[24] Chiou W.F., Lin L.C., Chen C.F. (2004). Acteoside protects endothelial cells against free radical-induced oxidative stress. J. Pharm. Pharmacol..

[25] Pastore S., Potapovich A., Kostyuk V., Mariani V., Lulli D., De Luca C., Korkina L. (2009). Plant polyphenols effectively protect HaCaT cells from ultraviolet C–triggered necrosis and suppress inflammatory chemokine expression. Ann. N. Y. Acad. Sci..

[26] Zeng J.-C., Fan Y.-G., Liu J.-R., Zeng Y.-R., Yi C.-Z., Yan L. (2010). Experimental study of directional differentiation of bone mesenchymal stem cells (BMSCs) to osteoblasts guided by serum containing cistanche deserticola. Zhongguo Gu Shang.

[27] Li T.M., Huang H.C., Su C.M., Ho T.Y., Wu C.M., Chen W.C., Fong Y.C., Tang C.H. (2012). Cistanche deserticola extract increases bone formation in osteoblasts. J. Pharm. Pharmacol..

[28] Xu X., Zhang Z., Wang W., Yao H., Ma X. (2017). Therapeutic Effect of Cistanoside A on Bone Metabolism of Ovariectomized Mice. Molecules.

[29] Lee S.Y., Lee K.S., Yi S.H., Kook S.H., Lee J.C. (2013). Acteoside suppresses RANKL-mediated osteoclastogenesis by inhibiting c-Fos induction and NF-kappaB pathway and attenuating ROS production. PLoS ONE.

[30] Bott K.N., Yumol J.L., Comelli E.M., Klentrou P., Peters S.J., Ward W.E. (2021). Trabecular and cortical bone are unaltered in response to chronic lipopolysaccharide exposure via osmotic pumps in male and female CD-1 mice. PLoS ONE.

[31] Chen Y., Liu S., Leng S.X. (2019). Chronic Low-grade Inflammatory Phenotype (CLIP) and Senescent Immune Dysregulation. Clin. Ther..

[32] Droke E.A., Hager K.A., Lerner M.R., Lightfoot S.A., Stoecker B.J., Brackett D.J., Smith B.J. (2007). Soy isoflavones avert chronic inflammation-induced bone loss and vascular disease. J. Inflamm..

[33] Smith B.J., Chongwatpol P., Rendina-Ruedy E., Stoecker B.J., Clarke S.L., Lucas E. (2015). Implications of compromised zinc status on bone loss associated with chronic inflammation in C57BL/6 mice. J. Inflamm. Res..

[34] Cao J.J., Gregoire B.R., Shen C.-L. (2017). A High-Fat Diet Decreases Bone Mass in Growing Mice with Systemic Chronic Inflammation Induced by Low-Dose, Slow-Release Lipopolysaccharide Pellets. J. Nutr..

[35] Smith B., Lerner M., Bu S., Lucas E., Hanas J., Lightfoot S., Postier R., Bronze M., Brackett D. (2006). Systemic bone loss and induction of coronary vessel disease in a rat model of chronic inflammation. Bone.

[36] Shen C.-L., Yeh J.K., Cao J.J., Tatum O.L., Dagda R.Y., Wang J.-S. (2010). Green tea polyphenols mitigate bone loss of female rats in a chronic inflammation-induced bone loss model. J. Nutr. Biochem..

[37] Shen C.-L., Yeh J.K., Samathanam C., Cao J.J., Stoecker B.J., Dagda R.Y., Chyu M.-C., Dunn D.M., Wang J.-S. (2011). Green tea polyphenols attenuate deterioration of bone microarchitecture in female rats with systemic chronic inflammation. Osteoporos. Int..

[38] Erel O. (2005). A new automated colorimetric method for measuring total oxidant status. Clin. Biochem..

[39] Alipieva K., Korkina L., Orhan I.E., Georgiev M.I. (2014). Verbascoside—A review of its occurrence, (bio)synthesis and pharmacological significance. Biotechnol. Adv..

[40] Cardinali A., Pati S., Minervini F., D’antuono I., Linsalata V., Lattanzio V. (2012). Verbascoside, Isoverbascoside, and Their Derivatives Recovered from Olive Mill Wastewater as Possible Food Antioxidants. J. Agric. Food Chem..

[41] Sendur O.F., Turan Y., Tastaban E., Serter M. (2009). Antioxidant status in patients with osteoporosis: A controlled study. Joint Bone Spine.

[42] Ray G., Husain S.A. (2002). Oxidants, antioxidants and carcinogenesis. Indian J. Exp. Biol..

[43] Fraser J., Helfrich M., Wallace H., Ralston S. (1996). Hydrogen peroxide, but not superoxide, stimulates bone resorption in mouse calvariae. Bone.

[44] Bánhegyi G., Csala M., Szarka A., Varsányi M., Benedetti A., Mandl J. (2003). Role of ascorbate in oxidative protein folding. BioFactors.

[45] Schröder K. (2014). NADPH oxidases in bone homeostasis and osteoporosis. Cell. Mol. Life Sci..

[46] Gowen L.C., Petersen D.N., Mansolf A.L., Qi H., Stock J.L., Tkalcevic G.T., Simmons H.A., Crawford D.T., Chidsey-Frink K.L., Ke H.Z. (2003). Targeted Disruption of the Osteoblast/Osteocyte Factor 45 Gene (OF45) Results in Increased Bone Formation and Bone Mass. J. Biol. Chem..

[47] David V., Martin A., Hedge A.-M., Rowe P.S.N. (2009). Matrix Extracellular Phosphoglycoprotein (MEPE) Is a New Bone Renal Hormone and Vascularization Modulator. Endocrinology.

[48] Donmez B.O., Karagur E.R., Donmez A.C., Choi J., Akkus O. (2022). Calcium-dependent activation of PHEX, MEPE and DMP1 in osteocytes. Mol. Med. Rep..

[49] Che C.-T., Wong M.S., Lam C.W.K. (2016). Natural Products from Chinese Medicines with Potential Benefits to Bone Health. Molecules.

